# Frontline Maintenance Treatment for Ovarian Cancer

**DOI:** 10.1007/s11912-021-01088-w

**Published:** 2021-06-14

**Authors:** Osnat Elyashiv, Yien Ning Sophia Wong, Jonathan A. Ledermann

**Affiliations:** 1 UCL Cancer Institute, University College CRUK and UCL Cancer Trials Centre, London, WC1E 6DD UK; 2 University College London Hospital, 235 Euston Rd, Fitzrovia, London, NW1 2BU UK

**Keywords:** Epithelial ovarian cancer, Fallopian tube cancer, Primary peritoneal cancer, Frontline maintenance treatment, Antiangiogenic agents, PARP inhibitors, Checkpoint inhibitors, Targeted therapies, Combined targeted therapies, Hormonal maintenance treatment

## Abstract

**Purpose of Review:**

Advanced epithelial ovarian cancer remains the most lethal gynaecological cancer. Most patients with advanced disease will relapse within 3 years after primary treatment with surgery and chemotherapy. Recurrences become increasing difficult to treat due to the emergence of drug resistance and 5-year survival has changed little over the last decade. Maintenance treatment, here defined as treatment given beyond primary chemotherapy, can both consolidate the response and prolong the control of disease which is an approach to improve survival.

**Recent Findings:**

Here we review maintenance strategies such as targeting angiogenesis, interference of DNA repair through inhibition of PARP, combinations of targeting agents, and immunotherapy and hormonal therapy.

**Summary:**

Much has been learnt from the success and challenges of these treatments that have in the last few years which led to significant reduction in disease recurrence, changed the guidelines for treatment, and established a new paradigm for the treatment of ovarian cancer.

## Introduction

Epithelial ovarian cancer (EOC) continues to be the leading cause of death from gynaecological malignancies. Worldwide, there are an estimated 295,000 cases and 184,000 ovarian cancer related-deaths documented in 2018[1]. Most patients present at advanced stage, which contributes to the high mortality rate. About 70% of all EOC are high grade serous tumours, most probably originating in the distal fallopian tube [2]. Other high-grade tumours of endometrioid or clear cell subtype are generally managed in the same way as the more common serous ovarian cancers. It is also now clear that a significant fraction of high grade EOC with non-mucinous histology, particularly the serous subtype, has an underlying hereditary cause. Around 20–25% of high grade serous EOC are associated with germline mutations in BRCA1/2 genes [3], and additional 5–7% of patients harbour somatic BRCA1/2 mutations [4]. Other rarer gene mutations, such as those associated with Lynch syndrome, are also found in association with ovarian cancers. The combination of cytoreductive surgical debulking followed by a platinum-based chemotherapy has been the mainstay of treatment for more than two decades. Maximal surgical effort resulting in no visible residual macroscopic disease yields the best outcome [5]. Despite good clinical response to primary treatment, the majority of patients with advanced stage disease experience disease relapse at 18 months [6]. Thereafter, the median survival is around 2 years [7].

The concept of maintenance treatment with further chemotherapy evolved from treatment of acute leukaemia and has also been applied to ovarian cancer. However, the success of this approach has not been established in solid tumours, including ovarian cancer. Prolonged therapy with, for example, low dose alkylating agents to consolidate the response and maintenance symptom (disease) control increases the risk of toxicity, including secondary leukaemia [8]. However, one trial in ovarian cancer conducted by the Southwest Oncology Group (SWOG) and Gynecologic Oncology Group (GOG) using maintenance paclitaxel for 12 or 3 months showed an improvement in progression-free survival. As a result, the trial was stopped by the Data Monitoring and Safety Committee but longer follow-up failed to show a survival advantage [9]. A similar trial by Pecorelli et al., assessing the addition of 6 cycles of paclitaxel after primary response to combination chemotherapy, failed to show benefit in PFS or OS [10]. In a novel approach, almost 20 years ago, maintenance subcutaneous interferon alpha or observation was explored in a randomised trial in women who had responded to chemotherapy and surgery. This trial also failed to show clinical benefit over standard of care [11].

It became clear that a maintenance strategy to sustain the benefit of primary treatment and prolong disease control and ultimately increase survival would depend on having a better understanding of the biology of ovarian cancer and developing approaches that would lead to a sustained process that targeted pathways involved in tumour growth and survival. In the last decade, a variety of strategies have emerged targeting tumour angiogenesis, DNA repair processes, and the host’s immune system. Some of these have resulted in significant benefit and are re-shaping the way in which ovarian cancer is now being treated.

## Anti-Angiogenesis

The role of angiogenesis is a well-known hallmark in cancer pathway [12]. In ovarian cancer, VEGF is known to play a role in peritoneal dissemination, tumour progression, and ascites formation [13, 14]. Two randomised trials, GOG 2018 and ICON7, explored the addition of bevacizumab, a monoclonal antibody that binds to VEGFA given with first-line chemotherapy and then as maintenance. The GOG 218 study was a three-arm placebo-controlled study in 1837 women with newly diagnosed, incompletely resected stage III-IV epithelial ovarian, tubal, or primary peritoneal cancer. Bevacizumab (15mg/kg) was added to the two experimental arms—one receiving bevacizumab only concurrently with chemotherapy, and another arm continuing as maintenance therapy for total of 21 cycles [15]. In the ICON7 trial, 1528 patients with newly diagnosed stages I–IV epithelial ovarian, tubal, or primary peritoneal cancer were randomised to receive standard carboplatin and paclitaxel chemotherapy or the addition of bevacizumab (7.5mg /kg), which was given concurrently with chemotherapy and continued every 3 weeks for total of 18 cycles [16]. The improvement in median PFS [15, 16] for both trials was about 2–4 months with the addition of bevacizumab. Additionally, a subgroup analysis in GOG 218 patients for whom increased CA125 result were censored, resulted in 6 months benefit compared to control group (12 vs 18 months, HR 0.645, 95% CI, 0.551 to 0.756) [15] and as a result, bevacizumab of 15 mg/kg was approved by the EMA and many regulatory authorities around the world. However, there was no improvement in overall survival which was one reason for the delay in approval by the FDA. A retrospective subgroup analysis of ICON7 showed a survival benefit for bevacizumab in women with a higher risk of recurrence (Stage III with ≥ 2cm residual disease/stage IV). The median OS was 39.7 versus 30.2 months (hazard ratio (HR) 0.78; 95% CI 0.63–0.97) [17]. In a similar analysis in stage IV patients treated in GOG 218, the median OS was 42.8 in the bevacizumab maintenance group compared to 32.6 months in the placebo-control arm ( HR 0.75; 95% CI 0.59–0.95) [18]. The treatment is generally well-tolerated with hypertension and proteinuria being the main side effects. Most patients were able to complete treatment, unless tumour progression occurred, and for many countries, bevacizumab, either at the full or half dose, was incorporated into treatment guidelines as a standard of care.

Three additional phase 3 trials have evaluated the efficacy of other anti-angiogenic agents. Two were with the oral broad-spectrum tyrosine kinase inhibitors (TKIs) that both targeted the VEGF receptor. The design of these studies was broadly similar; the pazopanib study was a switch maintenance trial after chemotherapy and the nintedanib included patients treated during chemotherapy and as maintenance. Patients with stages II–IV EOC were included and both trials showed an improvement in median PFS; for pazopanib, there was a median PFS benefit of 5.6 months (HR 0.77; 95% CI, 0.64–0.91; *p* = .0021) and for nintedanib it was 1.4 months (HR 0.84; 95% CI 0.72–0.98; *p* = 0.024), respectively [19, 20]. Final OS analysis of both trials did not show difference between treatment and placebo groups [21, 22]. While the HR for PFS in these studies was similar to bevacizumab, none of the oral tyrosine kinase inhibitors has been submitted for licensing for maintenance treatment in EOC.

The third trial, TRINOVA 3, evaluated the addition of trebananib, a peptibody that interferes with the angiopoietin pathway neutralising the interaction between Ang1 and Ang2 and the Tie2 receptor in patients with advanced EOC. Treatment group received weekly intravenous trebananib concurrently with frontline chemotherapy and continued as maintenance for up to 18 months. Median PFS did not differ between the trebananib group (15·9 months) and the placebo group (15·0 months) (HR 0·93; 95% CI 0·79–1·09; *p*=0·36) [23].

Potential candidate predictors of response to VEGF inhibitors identified in the GOG 218, such as CD31 immunohistochemistry, failed to show a correlation with a response to treatment [18]. Translational studies from a cohort within the ICON7 trial proposed a discriminatory signature comprising mesothelin, fms-like tyrosine kinase-4 (FLT4), α-1 acid glycoprotein (AGP), and cancer antigen 125 (CA-125) as potentially identifying those patients with EOC more likely to benefit from bevacizumab [24], as well as the potential role of Ang1 and Tie2 serum levels [25]. However, these markers need further validation in a broader group of patients from larger trials. The bevacizumab results met great enthusiasm which have been tempered by the results of the trials with oral anti-angiogenic agents, the lack of OS benefit from anti-angiogenic therapy, and perhaps more importantly the absence of predictive markers of benefit.

## Poly-(ADP-Ribose) Polymerase Inhibitors

The introduction of poly-(ADP-ribose) polymerase (PARP) inhibitors in the last decade has substantially changed the standard of care in the treatment of EOC. PARP enzymes are essential for the repair of single strand DNA breaks. Inhibition of PARP enzymes leads to an accumulation of double strand DNA breaks during replication and ultimately cell damage and death unless these DNA breaks can be repaired with high fidelity. Cancer cells with a BRCA mutation are deficient in the homologous recombination repair process (HRD), and are particularly sensitive to PARP inhibitors [26]. They are approved and widely used as maintenance therapy in platinum-responsive recurrent high-grade ovarian cancer following the publication of four trials with olaparib, niraparib, and rucaparib. HRD exists in a proportion of BRCA^wt^ tumours that respond to platinum-based therapy, and this has extended their use beyond tumours with a BRCA^mut^ [27–31]. As a consequence of these results, maintenance trials begun in the front-line setting, initially in patients with a BRCA^mut^. The first landmark in this area was the publication of the SOLO1 trial in which patients with stage III–IV high-grade serous or endometrioid ovarian, fallopian tube, or primary peritoneal cancer, with a germline or somatic BRCA1/2 mutation randomised to receive olaparib tablets or placebo for 24 months after completion of frontline platinum-based chemotherapy. The significant 70% reduction in the risk of disease progression or death in the olaparib arm (HR 0.30, 95% CI 0.23–0.41) had never been seen before in this setting, making the results of this trial an important cornerstone in the current practice of treatment in ovarian cancer [32••]. Recently presented updated data have shown that at 5 years, 48% of patients treated with olaparib remain free of progression compared with 21 % patients on placebo. The median PFS from the start of maintenance therapy is 56.0 months for olaparib versus 13.8 months in placebo (HR 0.33; 95% CI 0.25–0.430) [33••]. While OS data are not yet mature, these results underscore the value of using a PARP inhibitor as maintenance therapy in the first-line treatment of women with a BRCA mutation, and the importance of testing all patients for the presence of a germline or somatic BRCA mutation.

Recognising the value of PARP inhibitors in patients without BRCA mutations and with HRD in recurrent ovarian cancer, three further trials were conducted to explore the activity of PARP inhibitors in patients not selected for a BRCA mutation. Two of these trials had a switch maintenance design after first-line treatment with surgery and chemotherapy, adding niraparib or olaparib [34••, 35••] and the third included the PARP inhibitor, veliparib with chemotherapy and then as maintenance [36••]. In the PRIMA trial, patients with FIGO stage III or IV high-grade serous or endometrioid randomised to receive oral niraparib or placebo after demonstrating a response to platinum-based chemotherapy. A planned hierarchical analysis first included patients with homologous recombination deficiency, defined as either having a deleterious BRCA^mut^ or/a tumour HRD score, using the Myriad MyChoice test of at least 42, followed by the overall population. There followed an analysis in three subgroups: the BRCA^mut^/HRD positive; BRCA^wt^/HRD positive; and HR proficient groups. Significant prolongation of PFS seen with niraparib in all three groups, with diminishing benefit. The median PFS in each of the groups was, respectively, 22.1 months with niraparib versus 10.9 months with placebo (HR 0.43; 95% CI, 0.31 to 0.59; *p*<0.001), 19.6 versus 8.2 months (HR 0.50 (0.31–0.83)), and 8.1 versus 5.4 months (HR0.68 (0.49–0.94)) [34••]. As a result of the benefit in all three subgroups, niraparib maintenance is now widely licensed in all patients with stage III–IV high grade ovarian cancer.

A similar pattern of results was seen in the PAOLA-1 study in which patients received olaparib or placebo in addition to bevacizumab, given with chemotherapy and as maintenance. In addition to having two drugs during the maintenance phase, PAOLA-1 differed from PRIMA in that patients were not selected on the basis of a response to platinum-based therapy. Patients could enter if they were not progressing after primary treatment and included in this group were patients who had no evaluable disease after surgery (approximately 50% of the population). Similar benefits to PRIMA were seen among the HRD positive groups (using the Myriad assay), both with and without BRCA mutations but no benefit of adding olaparib to bevacizumab was seen in the HR proficient or unknown group. The median PFS in the three groups was 37.2 vs 17.7 months for patients with a tumour BRCA mutation (HR 0.33; 95% CI, 0.25 to 0.45); 28.1 vs 16.6 months in the HRD positive group without a BRCA mutation (HR 0.43; 95% CI, 0.28 to 0.66); and 16.9 vs 16.0 months in the HRD proficient or unknown group (HR 0.92; 95% CI, 0.72 to 1.17) [35••].

Earlier studies using either an oral VEGF receptor tyrosine kinase inhibitor (cediranib) or bevacizumab in combination with a PARP inhibitor have shown an additive or synergistic effect compared with PARP inhibitor [37, 38]. However, as PAOLA-1 did not have an olaparib alone arm, it was not possible to examine if there was any beneficial interaction between bevacizumab and olaparib. Nevertheless, the combination of olaparib maintenance and bevacizumab is now widely licensed for patients who have a HRD-positive test, with or without the presence of a BRCA mutation [39].

In the VELIA trial, veliparib tablets were given concurrently with chemotherapy to patients with high grade serous EOC and continued as maintenance in one of the 3 trial arms. The median PFS for patients receiving veliparib maintenance was again longer in all patient populations, but most beneficial in the BRCA^mut^ and the HRD group of patients compared to the control group, 34.7 vs 22 months (HR 0.44 (95% CI, 0.28–0.68); *p*<0.001) and 31.9 vs 20.5 months (HR, 0.57; 95% CI, 0.43 to 0.76; *p*<0.001), respectively [36••]. Table 1 summarises frontline PARP inhibitor maintenance treatment trials at frontline with comparisons of their PFS.

**Table 1:** First line maintenance treatment trials with PARP inhibitor

Trial name	Study group	Treatment arms	Median PFS for control group in months	Median PFS for treatment group in months (HR)
SOLO1	Stage III-IV high grade EOC with BRCA 1/2 mutation	Arm 1: Olaparib 300mg BD Arm 2: Placebo	13.8	49.9 (0.30)
PRIMA	Stage III-IV high grade EOC	Arm 1: Niraparib 300/200 mg OD Arm 2: Placebo	BRCA^mut^: 10.9 HRD: 8.2 HRP: 5.4	BRCA^mut^: 22.1 (0.40) HRD: 19.6 (0.50) HRP: 8.1 (0.68)
VELIA	Stage III-IV high grade serous EOC	Arm 1: Chemotherapy+ placebo followed with placebo maintenance Arm 2: Chemotherapy+ Veliparib followed by placebo maintenance Arm 3: Chemotherapy +Veliparib 150 mg, followed by Veliparib maintenance 400 mg BD	BRCA^mut^: 22.0 HRD: 30.5 Intention to treat: 7.3	BRCA^mut^: 37.7 (0.44) HRD: 31.9 (0.57) Intention to treat: 23.5 (0.68)
PAOLA1	Stage III-IV high grade EOC	Arm 1: Olaparib 300mg BD +Bevacizumab 15mg/kg q 3 weeks Arm 2: Placebo + Bevacizumab 15mg/kg q 3 weeks	Tumour BRCA^mut^: 21.7 Tumour BRCA -ve: 16.0 HRD (+ BRCA^mut^): 17.7 HRD (without BRCA): 16.6 HRP/unknown: 16.0	Tumour BRCA^mut^: 37.2 (0.31) Tumour BRCA -ve: 18.9 (0.71) HRD (+ BRCA^mut^): 37.2 (0.33) HRD (without BRCA): 28.1 (0.43) HRP/unknown: 16.9 (0.92)

*EOC*, epithelial ovarian cancer; *PFS*, progression-free survival; *HR*, hazard ratio; *HRD*, homologous recombination deficient; *HRP*, homologous recombination proficient

Adverse events are not uncommon in patients treated with PARP inhibitors, especially in the initial months of treatment and often require careful counselling and dose modifications. The most common adverse effects include fatigue, anaemia, and nausea. However, more severe toxicity (grade 3 and above) is uncommon, and the majority of patients are able to continue treatment after treatment interruptions and dose reductions [40, 41].

There is little doubt about the benefit in patients with a BRCA mutation, and there are no clear differences between the activity of olaparib or niraparib in this subgroup. The situation for BRCA^wt^ HRD-positive patients is a little more complicated. While the niraparib approval covers all patients in this group, the olaparib approval is with bevacizumab. Currently there is only one validated test for HRD, and more widely available tests are needed for simpler and cheaper access, and to clearly define this group. For patients with HR proficient tumours, there is greater uncertainty. While a benefit was apparent in the niraparib trial, no difference in outcome was seen when olaparib was added to bevacizumab. As pointed out, the entry criteria of patients in the PRIMA and PAOLA-1 trial are different, so that comparisons between the two trial outcomes should not be made. Clinicians, however, have the option of using niraparib in the HR proficient population. There is now widespread regulatory approval of PARP inhibitor maintenance therapy following first-line treatment. But it should be noted that longer-term outcome data for these patients is not yet available, and consideration needs to be given to whether all patients should receive maintenance with a PARP inhibitor at first-line or whether some women might derive greater benefit if these drugs are used in the setting of recurrent disease.

## Immunotherapy

Abagovomab is a murine anti-idiotypic antibody whose variable epitope mimics the tumour antigen (CA-125). This mouse monoclonal antibody presents CA-125 to the immune system to enhance the immune response leading to recognition and killing of tumour cells expressing CA-125. The MIMOSA study randomised in a 2:1 ratio nearly 900 patients with stage III to IV ovarian cancer in complete clinical remission after primary surgery and platinum- and taxane-based chemotherapy to abagovomab or placebo as maintenance therapy. However, maintenance abagovomab did not prolong recurrence free survival or OS [42].

It is now known that the immune system depends on various mechanisms, notably immune checkpoints, to maintain self-tolerance, prevent autoimmunity, and protect tissue from damage after activation of the immune response to pathogens. The immune system is heavily regulated through these checkpoint molecules, and this may explain why increased antigen presentation with abagovomab failed to generate a therapeutic immune response. Nevertheless, there are indicators to suggest that the tumour microenvironment in ovarian cancer is immunoreactive. Studies including a meta-analysis have shown that the presence of tumour-infiltrating lymphocytes (TILs) in ovarian cancer is associated with improved clinical outcome [43, 44]. Immunotherapy using checkpoint inhibitors (CPI), targeting CTLA-4, and/or PD-1/PD-L1 pathways has demonstrated a durable response in patients with certain solid tumours including melanoma, lung, bladder, and renal cancers. It is well established that CPI works by enhancing effector T cell responses, regardless of tumour type [45]. However, the results of studies with CPI in ovarian cancer have not thus far been encouraging. Early phase trials in advanced recurrent ovarian cancer have reported modest response rates of between 10 and 20%, with up to 45% disease control rate [46–48], compared to other solid tumour types.

Notably, JAVELIN Ovarian 100 trial (NCT02718417), the first randomised first-line maintenance phase III trial was terminated prematurely due to lack of efficacy following a planned interim futility analysis after all patients had been accrued. The trial included 998 newly diagnosed untreated stage III–IV patients due to start platinum-based chemotherapy. Patients were randomised to receive 3-weekly avelumab (an anti-PD-L1 antibody) with chemotherapy, followed by maintenance or after chemotherapy as maintenance therapy or chemotherapy alone. Bevacizumab was not given [49]. Before the interim results of JAVELIN Ovarian 100 were available, another randomised phase III trial, JAVELIN Ovarian PARP 100 trial (NCT03642132) with avelumab, was started. This included the PARP inhibitor, talazoparib, and the standard of care control arm was chemotherapy with bevacizumab. The experimental arms were avelumab with chemotherapy and as maintenance with maintenance talazoparib, and talazoparib alone [50]. In view of the interim results of JAVELIN 100, the trial was terminated with only a few patients accrued. This was unfortunate, as the design allowed for comparisons with chemotherapy and bevacizumab and would have also given information on talazoparib alone and the combination with avelumab. These negative results have recently been followed by those of IMagyn050/GOG 3015/ENGOT-OV39, a large phase III double-blind trial looking at a similar group of patients to JAVELIN Ovarian 100 trial. A total of 1301 women were randomised 1:1 to receive 3-weekly atezolizumab or placebo, with paclitaxel and carboplatin and then as maintenance for a total of 22 cycles. Bevacizumab was given to both arms for 22 cycles. After a median follow-up for 20 months, there was no statistically significant progression-free survival (PFS) improvement in either the intent-to-treat (ITT) population with a median of 18.4 months with placebo and 19.5 months with atezolizumab (HR 0.92; 95% CI 0.79–1.07) or the PD-L1+ population with a median of 18.5 vs 20.8 months respectively (HR 0.80; 95% CI 0.65–0.99). There was a trend towards a PFS benefit with atezolizumab in a subgroup with PD-L1 IC ≥5%. The interim overall survival (OS) did not show a benefit for atezolizumab, although the data are immature [51]. Overall, these two trials have shown that immune checkpoint inhibitors alone do not improve the outcome of patients undergoing first-line therapy for ovarian cancer. Much of the investment now is in studies combining immune checkpoint inhibitors with PARP inhibitors with or without additional bevacizumab.

## Combining Targeted Therapies

Understanding the factors responsible for generating tumour immunity and suppression is key to the success of immunotherapy in ovarian cancer, and it has been hypothesised that greater benefit can be achieved by combining different targeted therapies. Approximately 50% of high-grade serous ovarian cancer have homologous recombination deficiency including BRCA1 and/or BRCA2 mutations, with over 90% have TP53 mutations [52], and these mutations may lead to increased genetic instability, potentially increasing tumour immunogenicity. Moreover, BRCA-mutated and TP53-mutated ovarian cancer often contain increased number of tumour-infiltrating lymphocytes (TILs) and express PD-1/PD-L1 [53, 54]. Coupled with the presence of TILs in these patients, this suggests that CPI should be effective in patients with ovarian cancer. However, we now understand that all steps in the cancer immunity cycle are important to harness an anti-tumour immune response [55]. The interaction between the immune system and tumour antigens is a cyclical process involving tumour antigen recognition by the immune system with antigen presentation first and foremost, before priming and activation of the immune system. It is now widely accepted that ovarian cancers not only have low tumour mutational burden [56], but also low T cell-gene expression profiles in about 70–75% of patients [57]. Moreover, ovarian cancer is strongly dominated by copy number alterations [58], and it has been shown that some of these alterations evade the immune system in other tumour types like lung cancer [59, 60]. Moreover, tumour-associated macrophages (TAM), which have immunosuppressive effects, constitute a vital leucocyte population in ovarian cancer [61, 62]. Taken together, there are reasons why CPI, when used alone, has only yielded modest activity. It is crucial to re-examine the tumour microenvironment in ovarian cancer for changes pre- and post-treatment to help identify key changes to further increase efficacy of CPI [63–66]. In order to overcome these issues, several trials have been designed to enhance activation of the immune response by combining targeted therapies such as PARP and/or VEGF inhibitors. Many of these frontline maintenance trials are now underway and are summarised in Table 2. This has been a huge investment by the pharmaceutical industry, and a very large number of women have been enrolled in these trials. Also, these studies started before the results of the more recent PARP inhibitor studies which included BRCA^wt^ patients became available. Whatever the outcome, it may be difficult to show that CPI combined with PARP inhibitors with or without bevacizumab augment the results we already have with PARP inhibitors alone or with bevacizumab.

**Table 2:** Phase III first-line trials with PARP inhibitors ± VEGF inhibitors in combination with an immune checkpoint inhibitor (CPI)

Trial name	Trial details (number, completion date)	PARP-inhibitor	Immune checkpoint inhibitor	Chemotherapy	Maintenance
ATHENA (NCT03522246)	*N* = 1000 December 2024 Recruitment stopped	Rucaparib	Nivolumab	Standard of care (not part of trial)	1. Nivolumab + Rucaparib 2. Nivolumab placebo + Rucaparib 3. Nivolumab + Rucaparib placebo 4. Placebo + Placebo
DUO-O (NCT03737643)	*N* = 1254 June 2023 Still recruiting	Olaparib	Durvalumab	Somatic BRCA wildtype: 1. CT + Bev + placebo 2. CT + Bev + Durva 3. CT + Bev + Durva Somatic BRCA mutant: 4. CT + Bev (optional) + Durva	tBRCA wildtype: 1. Bev + Durva placebo + Olaparib placebo 2. Bev + Durva + Olaparib placebo 3. Bev + Durva + Olaparib tBRCA mutant: 4. Bev (optional) + Durva + Olaparib
MK-7339-001/KEYLYNK-001/ENGOT-ov43/GOG-3036 (NCT03740165)	*N* = 1086 August 2025 Still recruiting	Olaparib	Pembrolizumab	1. CT + pembrolizumab 2. CT + pembrolizumab 3. CT + placebo	1. Pembro + Olaparib 2. Pembro + placebo 3. Placebo + placebo
ENGOT-0V44 FIRST trial (NCT03602859)	*N* = 1228 February 2023 Still recruiting	Niraparib	TSR-042 (Dostarlimab)	1. CT + TSR-042 2. CT 3. CT	1. Niraparib + TSR-042 2. Niraparib 3. Placebo

*Bev*, bevacizumab; *CT*, chemotherapy; *Durva*, durvalumab; *N*, number

## Hormonal Maintenance Therapy

Immunohistochemistry demonstrates estrogen receptor (ER) in 43–81% of ovarian cancers [67]. Highest ER positivity has been reported in low-grade serous ovarian cancer (LGSOC), followed by high-grade endometrioid and serous EOC [68]. As LGSOC is known to have a more indolent course and a poorer response to standard chemotherapy, there has been much interest in exploring the possible clinical significance of the high percentage of ER and progesterone receptor (PR) positivity in these tumours. ER positivity in LGSOC has been found to be an independent prognostic variable for improved survival [67, 69] and treatment with tamoxifen, or aromatase inhibitors has shown clinical benefit in relapsed HGSOC. A study by Gershenson et al. demonstrated significantly longer PFS in a retrospective series of patients with LGSOC after primary surgery followed by platinum-based chemotherapy who received hormone maintenance treatment versus observation (64.9 vs 26.4 months, *p*< .001, respectively) [70]. Although randomised data are lacking in LGSOC, there is a generally held view that adjuvant chemotherapy has little benefit, so letrozole is being increasingly adopted as a standard after surgery. Formal testing is being undertaken in an NRG and National Cancer Institute (NCI) study in a phase III trial to assess non-inferiority of letrozole monotherapy versus platinum-based chemotherapy followed by maintenance letrozole for patients with newly diagnosed stage II–IV LGSOC after primary cytoreductive surgery. The results of this trial may shed more light on the treatment of LGSOC in the frontline setting (NCT04095364).

## Conclusion

The paradigm of maintenance treatment in EOC has shifted largely during the last decade with the introduction of targeted treatments in the frontline maintenance setting. Despite variations in drug accessibility in different countries maintenance, therapy has become a standard of care for the treatment of advanced EOC. The use of bevacizumab has become widespread and the newer highly positive results of first-line PARP inhibitor trials underscore the importance for biomarker-driven therapy. Testing for a BRCA mutation is now regarded as a standard investigation in patients with high grade EOC. The encouraging maturing results of PARP inhibitor maintenance in BRCA-mutated ovarian cancer provides optimism for a wider use of these drugs in HRD deficient tumours, particularly when testing for genomic instability becomes more readily available and widespread. Progress in bringing CPI into the treatment of ovarian cancer has been more challenging but the results of important trials combining CPI and PARP inhibitors will soon be available. It is becoming increasingly clear that the maintenance therapy is becoming the norm for first-line treatment, but this also opens up challenges for treating women who relapse after these treatments. More research is needed to see whether retreatment with PARP inhibitors or bevacizumab is possible, either alone, together, or in combination with other novel agents.
